# Clinicopathological spectrum of biopsy-proven renal diseases of patients at a single center in Sri Lanka: a cross sectional retrospective review

**DOI:** 10.1186/s12882-023-03217-y

**Published:** 2023-06-22

**Authors:** Chanaka Muthukuda, Vindika Suriyakumara, Cherine Sosai, Thilina Samarathunga, Maithili Laxman, Arjuna Marasinghe

**Affiliations:** 1 Nephrology and dialysis unit, Colombo South Teaching Hospital, Kalubowila, Sri Lanka; 2 Ministry of Health and Indigenous Medical Services, Colombo, Sri Lanka; 3 Histopathology unit, Colombo South Teaching Hospital, Kalubowila, Sri Lanka; 4 Diabetes Research Unit, Department of Clinical Medicine, Faculty of Medicine, University of Colombo, Colombo, Sri Lanka

**Keywords:** Renal biopsy, Glomerulonephritis, Nephrotic range proteinuria, Hematuria, South Asia

## Abstract

**Background:**

The clinical presentation of renal diseases can vary widely. The lack of a comprehensive national registry for Sri Lanka makes it difficult to provide a detailed record of the various clinical presentations and histopathology of renal disorders in the nation. Therefore, this study aims to provide a record of the spectrum of renal diseases in Sri Lanka.

**Methods:**

Renal biopsies performed at the nephrology unit in Colombo South Teaching Hospital (CSTH), Sri Lanka from March 2018 to October 2019 was retrospectively studied. Indications for renal biopsy were nephrotic range proteinuria, sub nephrotic range proteinuria, acute kidney injury without obvious etiology, chronic renal disease without obvious etiology and haematuria.

**Results:**

A total of 140 native kidney biopsies were analyzed in which majority were females (55.7%). The mean age of the population was 46 ± 15.3 years. The most common indications for renal biopsy were nephrotic range proteinuria (54.3%), followed by sub-nephrotic range proteinuria (14.3%), nephrotic range proteinuria with haematuria (14.3%), sub-nephrotic range proteinuria with haematuria (9.3%), AKI without known cause (4.3%), and CKD without known cause (3.6%). The leading histopathological diagnoses were FSGS (22.1%), lupus nephritis (20%), PSGN (17.1%), DN (12.1%), HTN (9.3%), MCD (6.4%), IgA nephropathy (5.7%), IN (4.3%), vasculitis (2.1%), and MGN (0.7%).

**Conclusions:**

The most common indication for renal biopsy was nephrotic range proteinuria in our population. FSGS was the most prevalent histopathological diagnosis and the least frequent diagnosis reported was MGN. The spectrum of renal diseases could differ according to the study location and it changes over time. Therefore, a renal biopsy registry is needed for documenting the changing disease pattern in Sri Lanka.

## Background

End-stage renal disease has become a growing burden especially in the developing nations and recent studies indicate that the number of patients starting kidney replacement therapy due to glomerular diseases is on the rise [1]. Renal biopsy has become a standard tool for diagnosis of renal parenchymal diseases and has a high accuracy in prognosis and treatment. The first publication on renal biopsy was in 1951 by Iversen and Brun which paved for understanding of the pathogenesis and diagnosis of kidney disease [2]. Over the years with the development of immunohistochemistry, immunofluorescence and electron microscopy, renal biopsy technique improved its diagnostic potential providing more information on the histopathology and classification of kidney diseases [3, 4].

Renal disorders are of many subtypes, glomerulonephritis (GN) is the most frequent type of renal disorder and its epidemiology is mainly determined by the biopsy rate [4, 5]. Epidemiology of biopsy proven renal diseases is important to understand the geographical prevalence and variability of renal diseases. Previous studies show that immunoglobulin A nephropathy (IgAN) was the most common cause of GN in Japan [6], China [7, 8], Australia [9], Hungary [10], Italy [11, 12], Spain [13] and France [14]. Focal and segmental glomerulosclerosis (FSGS) was reported as the most frequent cause of GN in India [15–18], Pakistan [19] Brazil [20], Colombia [21] and USA [22]. These varying reports from different parts of the world could be influenced by many confounding factors including socioeconomic status, geography, ethnicity, time period, nutritional status and age [4, 6].

There is limited data regarding the patterns of renal diseases in Sri Lanka, and a centralised national registry is unavailable for renal diseases in Sri Lanka. Therefore, the aim of this study was to provide a comprehensive record of the clinicopathological spectrum of renal diseases according to clinical presentation and histopathological diagnoses and emphasize the importance of maintaining a central national database/registry for renal diseases in Sri Lanka.

## Methods

### Study design

Clinical and pathological records of adult patients (≥18 years) who had a native kidney biopsy performed at the nephrology unit in Colombo South Teaching Hospital (CSTH), Sri Lanka during the period of March 2018 to October 2019 were retrospectively reviewed. Colombo South Teaching Hospital is one of the leading tertiary care centers in Sri Lanka and patients with renal diseases are referred to the nephrology team lead by the consultant nephrologist. If indications are sufficient, the consultant nephrologists would direct the patients for renal biopsy which are being performed under real time ultra sound scan guidance. Renal biopsy was not performed on patients who had diabetes mellitus with diabetic retinopathy unless there was a compelling indication.

### Indications for renal biopsy

Indications for biopsy were defined as follows:

- Acute kidney injury without obvious etiology (AKI) – Increase in serum creatinine by >0.3mg/dl (>26.5 mmol/l) within 48 hours; or increase in serum creatinine >1.5 times of baseline, which is known or presumed to have occurred within the prior 7 days; or urine volume <0.5ml/kg/h for 6 hours without a known etiology [23].
- Chronic renal disease without obvious etiology (CKD) – Abnormality of kidney functions or structure present for 3 months without obvious etiology with normal kidney sizes were not contraindications for biopsy [24].
- Nephrotic range proteinuria- Defined by 24-hr urine protein > 3 g/day/1.73 m^2^, UPCR > 300mmol/l with or without edema [25].
- Sub nephrotic range proteinuria-Defined by 24-hr urine protein between 1-3 g/day/1.73 m^2^, Urine Protein Creatinine Ratio (UPCR) between 100-300mmol/l.
- Haematuria – Microscopic presence of red cells in urine full report together with the presence of proteinuria (Hematuria alone was not an indication for renal biopsy).

### Diagnosis criteria for hypertensive nephropathy (HTN).

The criteria for the diagnosis of hypertensive nephropathy were as follows: i) >5 year history of primary hypertension that is typically accompanied by left ventricular hypertrophy, coronary heart disease, heart failure, cerebral arteriosclerosis and/or history of cerebral vascular accident; ii) relatively normal urine sediment; iii) retinal arteriosclerosis or arteriosclerotic changes in the retina; iv) slowly progressive renal insufficiency with gradually increasing proteinuria that is usually non-nephrotic; v) hypertension precedes the development of either proteinuria or renal insufficiency and there is no other obvious cause of renal disease [26].

### Pathologic diagnosis

Two cores of kidney tissues were obtained in each case. One sample was sent for light microscopic study which was performed by the histopathologist at CSTH and second sample was sent to Medical Research Institute (MRI), Colombo, Sri Lanka for immunofluorescence. Then the first sample was fixed with 10% neutral buffered formal saline, routinely processed and embedded in paraffin wax, stained with haematoxylin and eosin (H&E) stain, Masson’s Trichrome, periodic acid–Schiff and Silver stains. Congo red stains was performed if required. The second sample was frozen sectioned for immunofluorescence examination of IgG, IgA, IgM, C3, C1q and fibrinogen. In CSTH Electron Microscope (EM) was not available, therefore the final most likely diagnosis was formulated based on clinicopathologic correlations of each patient.

### Data collection and analysis

The information was retrieved from the histopathology requisition forms accompanying renal biopsy and clinic data. We included histopathology requisition forms of patients who had undergone renal biopsies during the study period and who had sufficient samples for histopathological diagnosis. We excluded records with insufficient samples and hence had no histopathological diagnosis. The relevant clinical and laboratory variables such as socio-demographic data, indication for renal biopsy, histopathological diagnosis and clinical investigations were documented. The data was entered in Microsoft Office Excel spread sheet and statistical analysis was done using Statistical Analysis System (SAS) 9.4.

## Results

During the study period a total of 140 native kidney biopsies were performed at the center. There were 78 (55.7%) females and 62 (44.3%) males. The mean age of the sample was 46 ±15.3 years. The indication for renal biopsy and histopathological diagnoses are presented in Table 1. In both genders, the most common indications for renal biopsy were nephrotic range proteinuria amounting to 54.3%, followed by sub-nephrotic range proteinuria (14.3%), nephrotic range proteinuria with haematuria (14.3%), sub-nephrotic range proteinuria with haematuria (9.3%), AKI without known cause (4.3%), and CKD without known cause (3.6%). The leading histopathological diagnoses were FSGS (22.1%), lupus nephritis (20%), PSGN (17.1%), DN (12.1%), HTN (9.3%), MCD (6.4%), IgA nephropathy (5.7%), IN (4.3%), vasculitis (2.1%), and MGN (0.7%). The most common diagnoses in the nephrotic range proteinuria were FSGS accounting for 27.6% followed by DN (17.1%), lupus nephritis (14.5%), MCD (11.8%), IgA nephropathy (9.2%) and HTN (7.8%).

**Table 1:** Indication for renal biopsy and histopathological diagnoses

**Indication for biopsy**	**Histopathology Diagnoses**										**Total** **n**
	**DN**	**FSGS**	**HTN**	**IgA**	**IN**	**Lupus**	**MCD**	**MGN**	**PSGN**	**Vasculitis**	
**AKI without known cause n (%)**	0 (0)	1 (16.6)	0 (0)	1 (16.6)	1(16.6)	1 (16.6)	0(0)	0(0)	1(16.6)	1(16.6)	**6**
**CKD without known cause n (%)**	1 (20)	2 (40)	1 (20)	0 (0)	1(20	0 (0)	0(0)	0(0)	0(0)	0 (0)	**5**
**Nephrotic range proteinuria n (%)**	13 (17.1)	21 (27.6)	6 (7.8)	7 (9.2)	1(1.3)	11 (14.5)	9(11.8)	1(1.3)	6(7.8)	1 (1.3)	**76**
**Nephrotic range proteinuria with hematuria n (%)**	1 (5)	5 (25)	2 (10)	0 (0)	0 (0)	5 (25)	0 (0)	0(0)	6(30)	1 (5)	**20**
**Sub-Nephrotic range proteinuria n (%)**	2 (10)	1 (5)	4 (20)	0 (0)	3(15)	8 (40)	0 (0)	0(0)	2(10)	0 (0)	**20**
**Sub-Nephrotic range proteinuria with hematuria n (%)**	0 (0)	1 (7.7)	0 (0)	0 (0)	0(0)	3 (23.1)	0 (0)	0(0)	9(69.2)	0 (0)	**13**
**Total n (%)**	**17 (12.1)**	**31 (22.1)**	**13 (9.3)**	**8 (5.7)**	**6 (4.3)**	**28 (20)**	**9 (6.4)**	**1 (0.7)**	**24 (17.1)**	**3 (2.1)**	**140**

Renal biopsy indications for different age groups are shown in Table 2. Nephrotic range proteinuria was the most common indication for renal biopsy in all age groups. The mean age of the patients with nephrotic range proteinuria was 46.8 ±15.1 years. Majority of the patients who underwent biopsy were above 40 years. However, most patients presented with sub-nephrotic range proteinuria with haematuria were young adults under 40 years (69.2%).

**Table 2:** Renal biopsy indication in different age groups

**Indication for biopsy**	**Age (years)**	**16-30**	**30 ≤ age < 40**	**40 ≤ age < 50**	**50 ≤ age < 60**	**≥ 60**	**Total**
	**Mean**	***n* (%)**	***n* (%)**	***n* (%)**	***n* (%)**	***n* (%)**	***n* (%)**
**AKI without known cause**	48.1	1 (4)	0 (0)	1 (2.7)	3 (8.8)	1 (3.4)	6 (4.2)
**CKD without known cause**	58.2	0 (0)	0 (0)	1 (2.7)	1 (2.9)	3 (10.3)	5 (3.5)
**Nephrotic range proteinuria**	46.8	13 (52)	8 (50)	21 (58.3)	19 (55.8)	15 (51.7)	76 (53.5)
**Nephrotic range proteinuria with haematuria**	47.5	2 (8)	3 (18.7)	6 (16.6)	4 (11.8)	5 (17.2)	20 (14.1)
**Sub-nephrotic range proteinuria**	44.7	4 (16)	1 (6.2)	6 (16.6)	7 (20.6)	2 (6.9)	20 (14.1)
**Sub-nephrotic range proteinuria with haematuria**	36.8	5 (20)	4 (25)	1 (2.7)	0 (0)	3 (10.3)	13 (9.3)
**Total (n)**		**25**	**16**	**36**	**34**	**29**	**140**

Table 3 presents the disease pattern across different age groups. The most common diagnoses in the 16-30 years age category were lupus and PSGN whereas FSGS and HTN nephropathy were the most common diseases among the elderly (≥ 60 years). Majority of the diagnoses were found to be distributed in the age groups of above 40 years. Diabetes nephropathy was common in 50-60 years age group. IgA nephropathy was commonest in 16-30 years age group whereas MCD was almost similarly common in 16-30 years and 40-50 years age groups.

**Table 3:** Disease pattern in different age groups

**Histopathology Diagnoses**	**Age (years)**	**16-30**	**30 ≤ age <40**	**40 ≤ age <50**	**50 ≤ age <60**	**≥ 60**	**Total**
	**Mean**	***n* (%)**	***n* (%)**	***n* (%)**	***n* (%)**	***n* (%)**	***n***
**DN**	52.1	0 (0)	1 (6.2)	5 (13.8)	8 (24.2)	3 (10.3)	17
**FSGS**	51.9	1 (3.8)	4 (25)	9 (25)	7 (21.2)	10 (34.5)	31
**HTN**	58.2	0 (0)	1 (6.2)	1 (2.7)	5 (15.2)	6 (20.7)	13
**IgA**	40.3	3 (11.5)	1 (6.2)	1 (2.7)	2 (6.0)	1 (3.4)	8
**IN**	61.6	0 (0)	0 (0)	1 (2.7)	2 (6.0)	3 (10.3)	6
**Lupus nephritis**	36.5	10 (38.46)	5 (31.2)	8 (22.2)	4 (12.1)	1 (3.4)	28
**MCD**	40.5	3 (11.5)	0 (0)	4 (11.1)	1 (3.0)	1(3.4)	9
**MGN**	56	0 (0)	0 (0)	0 (0)	1 (3.0)	0 (0)	1
**PSGN**	39.4	9 (34.6)	4 (25)	5 (13.8)	3 (9.0)	3(10.3)	24
**Vasculitis**	49	0 (0)	0 (0)	2 (5.5)	0 (0)	1 (3.4)	3
**Total (n)**	**-**	**26**	**16**	**36**	**33**	**29**	**140**

Descriptive characteristics of indications for renal biopsy are presented in Table 4. Clinical details revealed 56 (40%) patients to have hypertension and 36 (25.7%) with diabetes mellitus without diabetic retinopathy. In all cases the bilateral kidney size was equal or more than 10 cm. Ultrasound scan had features of chronic renal parenchymal disease in 40 (28.6%) patients. Urinalysis indicated majority of the patients with protein 3+ (57.1%) and red cell casts in 4 (2.9%), granular casts in 32 (22.9%) and 16 (11.4%) patients had dysmorphic red blood cells ≥10%. Patients with nephrotic range proteinuria had the highest mean urine protein creatine ratio (UPCR) of 672.8 ± 363.7 mmol/l, whereas sub nephrotic range proteinuria had the lowest mean UPCR of 166.6 ± 65.8 mmol/l. The mean serum creatinine was highest among those with AKI of unknown cause (562.8 ± 377 mmol/l) and was followed by patients with CKD of unknown cause (450 ± 431.6 mmol/l). Those biopsied for nephrotic range proteinuria reported to have the lowest mean serum creatinine value (88.6 ± 61.7mmol/l).

**Table 4:** Descriptive characteristics of indications for renal biopsy

**Parameter**	**Indication for biopsy**						**Total**
	**AKI without known cause**	**CKD without known cause**	**Nephrotic range proteinuria**	**Nephrotic range proteinuria + hematuria**	**Sub-Nephrotic range proteinuria**	**Sub-Nephrotic range proteinuria + hematuria**	
**Total n**	**6**	**5**	**76**	**20**	**20**	**13**	**140**
**Gender**							
Male n (%)	4 (6.4)	3 (4.8)	33 (53.2)	10 (16.1)	6 (9.6)	6 (9.6)	**62**
Female n (%)	2 (2.5)	2 (2.5)	43 (54.4)	10 (12.6)	14 (17.7)	7 (8.8)	**78**
**Hypertension**							
Yes n (%)	4 (7.1)	2 (3.5)	33 (58.9)	9 (16)	6 (10.7)	2 (3.5)	**56**
No n (%)	2 (2.3)	3 (3.5)	43 (51.1)	11 (13)	14 (16.6)	11 (13)	**84**
**Diabetes mellitus**							
Yes n (%)	1 (2.8)	3 (8.3)	21 (58.3)	3 (8.3)	6 (16.6)	2 (5.5)	**36**
No n (%)	5 (4.8)	2 (1.9)	55 (52.8)	17 (16.3)	14 (13.4)	11 (10.5)	**104**
**Ultrasound Scan CKD**							
Yes n (%)	0 (0)	4 (10)	26 (65)	6 (15)	3 (7.5)	1 (2.5)	**40**
No n (%)	6 (6)	1 (1)	50 (50)	14 (14)	17 (17)	12 (12)	**100**
**UFR/Protein**							
Nil	2 (66.6)	0 (0)	0 (0)	0 (0)	0 (0)	1 (33.3)	**3**
1+ n (%)	1 (10)	1 (10)	0 (0)	0 (0)	6 (60)	2 (20)	**10**
2+ n (%)	1 (4.3)	1 (4.3)	2 (8.7)	1 (4.3)	10 (43.4)	8 (34.7)	**23**
3+ n (%)	1 (1.2)	3 (3.7)	56 (70)	14 (17.5)	4 (5)	2 (2.5)	**80**
4+ n (%)	1 (4.1)	0 (0)	18 (75)	5 (20.8)	0 (0)	0 (0)	**24**
**UFR/ RED Cell Cast**							
Yes n (%)	1 (25)	0 (0)	3 (75)	0 (0)	0 (0)	0 (0)	**4**
No n (%)	5 (3.6)	5 (3.6)	73 (53.6)	20 (14.7)	20 (14.7)	13 (9.5)	**136**
**UFR /Granular Cast**							
Yes n (%)	2 (6.2)	1 (3.1)	20 (62.5)	5 (15.6)	1 (3.1)	3(9.3)	**32**
No n (%)	4 (3.7)	4 (3.7)	56 (51.8)	15(13.8)	19(17.5)	10(9.2)	**108**
**UFR/Dysmorphic RBC**							
≥10% n (%)	2(12.5)	0 (0)	4 (25)	4 (25)	1 (6.2)	5(31.5)	**16**
<10% n (%)	4(3.2)	5 (4)	72 (58)	16(12.9)	19 (15.3)	8 (6.4)	**124**
**UPCR (mg/mmol)**							
Mean	358.2	560	672.8	633	167	249.6	
SD	281	196.3	363.7	274	65.8	288	
**Left Kidney size(cm)**							
Mean	10	10.7	10.3	10.1	10.1	10.6	
SD	1.3	1.5	1	1.2	0.6	0.9	
**Right Kidney size(cm)**							
Mean	9.7	10	10.1	10	10.2	10.7	
SD	1.4	2	1.1	1.1	0.7	0.9	
**Creatinine(mg/dl)**							
Mean	562.8	450	88.6	92.4	98.1	101	
SD	377	431.6	61.7	37.4	42.5	36.2	

Among the patients diagnosed with FSGS, 9 patients were known to have DM, 12 had HTN, 27 patients had 3+ and 4+ proteinuria and no one had red cell cast in urine (Table 5). In patients diagnosed with lupus, 4 patients had DM, 4 had HTN and 24 patients had 2+ and 3+ proteinuria. Out of the 24 patients diagnosed with PSGN only 2 patients had DM, 3 had HTN and 19 patients had 2+ and 3+ proteinuria. Among the 17 diabetic nephropathy patients, all had been previously diagnosed with DM and 15 also had HTN and 12 had 3+ proteinuria. From the 13 HTN nephropathy patients, all had been previously diagnosed with hypertension and 3 patients also diagnosed with diabetes, 9 patients had 3+ proteinuria.

**Table 5:** Descriptive characteristics of renal diseases

**Parameter**	**Histopathology diagnosis**										**Total**
	**DN**	**FSGS**	**HTN**	**IgA**	**IN**	**Lupus**	**MCD**	**MGN**	**PSGN**	**Vasculitis**	
**Total n**	**17**	**31**	**13**	**8**	**6**	**28**	**9**	**1**	**24**	**3**	**140**
**Gender**											
**Male n (%)**	14 (22.5)	13 (20.9)	7 (11.2)	2 (3.2)	4 (6.4)	0 (0)	2 (3.2)	1 (1.6)	16 (25.8)	3 (4.8)	62
**Female n (%)**	3 (3.8)	18 (23)	6 (7.6)	6 (7.6)	2 (2.5)	28 (35.8)	7 (8.9)	0 (0)	8 (10.2)	0 (0)	78
**HTN**											
**Yes n (%)**	15(26.7)	12(21.4)	13(23.2)	5(8.9)	2(3.5)	4(7.1)	2(3.5)	0 (0)	3(5.3)	0 (0)	56
**No n (%)**	2 (2.3)	19(22.6)	0 (0)	3 (3.5)	4(4.7)	24(28.5)	7(8.3)	1(1.1)	21(25)	3(3.5)	84
**DM**											
**Yes n (%)**	17(42.2)	9(25)	3(8.3)	1(2.7)	0 (0)	4 (11.1)	0 (0)	0 (0)	2 (5.5)	0 (0)	36
**No n (%)**	0 (0)	22(21.1)	10(9.6)	7(6.7)	6(5.7)	24(23)	9(8.6)	1(0.9)	22(21.1)	3(2.8)	104
**CKD Scan**											
**Yes n (%)**	10(25)	12(30)	8(20)	3(7.5)	2(5)	0 (0)	2 (5)	1 (2.5)	2(5)	0 (0)	40
**No n (%)**	7 (7)	19(19)	5 (5)	5 (5)	4 (4)	28(28)	7(7)	0(0)	22(22)	3 (3)	100
**UFR/Protein**											
**Nil**	0 (0)	0(0)	0 (0)	0 (0)	0 (0)	0 (0)	1 (33.3)	0 (0)	2 (66.6)	0 (0)	3
**1+ n(%)**	2 (20)	1(10)	1 (10)	0 (0)	2 (20)	3 (30)	0 (0)	0 (0)	1 (10)	0 (0)	10
**2+ n(%)**	1 (4.3)	3 (13)	1 (4.3)	0 (0)	1 (4.3)	8(34.7)	1 (4.3)	0 (0)	8 (34.7)	0 (0)	23
**3+ n(%)**	12(15)	15(18.7)	9(11.2)	6(7.5)	3(3.7)	16(20)	6(7.5)	1(1.2)	11(13.7)	1 (1.2)	80
**4+ n(%)**	2 (8.3)	12(50)	2 (8.3)	2(8.3)	0 (0)	1 (4.1)	1 (4.1)	0 (0)	2(8.3)	2 (8.3)	24
**UFR/ RED Cell Cast**											
**Yes n (%)**	1 (20)	0 (0)	0 (0)	0 (0)	0 (0)	1 (20)	1 (20)	0 (0)	2(40)	0 (0)	5
**No n (%)**	16(11.8)	31(22.9)	13(9.6)	8(5.9)	6(4.4)	27(20)	8(5.9)	1(0.7)	22(16.2)	3(2.2)	135
**UFR/Granular Cast**											
**Yes n (%)**	7(21.8)	7(21.8)	4(12.5)	2(6.2)	0 (0)	4 (12.5)	1 (3.2)	0 (0)	7 (21.8)	0 (0)	32
**No n (%)**	10(9.2)	24(22.2)	9(8.3)	6(5.5)	6(5.5))	24(22.2)	8(7.4)	1(0.9)	17(15.7)	3(2.7))	108
**UFR/Dysmorphic RBC**											
**≥10% n(%)**	2(12.5)	2(12.5)	2(12.5)	1(6.2)	0 (0)	4(25)	0 (0)	0 (0)	5(31.2)	0 (0)	16
**<10% n(%)**	15(12)	29(23.3)	11(8.8)	7(5.6)	6(4.8)	24(19.3)	9(7.2)	1(0.8)	19(15.3)	3(2.4)	124
**UPCR**											
**Mean**	669	606.5	524.6	694	378	389.4	826.3	413.4	431	569.5	
**SD**	359.4	459.8	349.9	358	259	254.2	364.2	.	306	156.8	
**Left Kidney size**											
**Mean**	10.5	9.8	9.9	10	10.3	10.4	10.6	8.9	10.4	10.7	
**SD**	1.1	0.9	1.3	0.9	1.2	0.9	1.5	.	0.9	0.7	
**Right Kidney size**											
**Mean**	10.4	9.7	9.8	9.6	10.3	10.5	9.5	8.3	10.3	11.4	
**SD**	0.98	0.8	1.2	1.2	1.1	1.3	1.4	.	0.9	0.5	

## Discussion

The present study was conducted to determine the epidemiology and the relationship between indication and histopathology outcomes of renal biopsies at a single center in Sri Lanka, which thereby would improve the existing clinical knowledge of possible cause of renal disorders in the nation. Majority of the patients in our cohort were females (55.7%) and the mean age was 46 ±15.3 years. The most common indication for biopsy was nephrotic range proteinuria (54.3%), followed by sub-nephrotic range proteinuria (14.3%), nephrotic range proteinuria with hematuria (14.3%), sub-nephrotic range proteinuria with hematuria (9.3%), AKI without known cause (4.3%) and CKD without known cause (3.6%) (Table 1). Nephrotic range proteinuria was the most common indication in all age groups and in both genders (Table 2).

The epidemiology of primary and secondary glomerular diseases may differ within the nation due to different socio-economic and ethnic backgrounds in different parts of the country. Similar to our findings, single center studies conducted in Sri Lanka showed nephrotic range proteinuria as the most common indication for renal biopsy [27, 28]. Comparably studies done in other parts of the world including Bangladesh [29], Africa [30], India [15], Poland [31], China [32] and Romania [33, 34] showed nephrotic range proteinuria as the most prevalent indication for renal biopsy.

The most frequent histopathological diagnosis in our study was FSGS (22.1%) followed by lupus nephritis (20%), PSGN (17.1%), DN (12.1%), HTN (9.3%), MCD (6.4%), IgA nephropathy (5.7%), IN (4.3%), Vasculitis (2.1%), and MGN (0.7%). Interestingly, primary GN was the most common finding and IgA nephropathy being the most frequent diagnosis in the study performed at Sri Jayewardenepura General Hospital, Sri Lanka between 2012 and 2019 [27]. However, this does not correspond with our data, which showed FSGS to be the leading cause. The second common histology in that study was FSGS followed by primary chronic TIN. Whereas in the secondary forms of GN, lupus was the most common followed by DN and amyloidosis [27]. Another retrospective study done at Kandy teaching hospital Sri Lanka with a total of 2680 biopsies during the period of 2010 to 2019 showed that among primary GN, FSGS as the most common diagnosis observed in 11.41% biopsies [28]. Among secondary GN, lupus nephritis was the most common diagnosis observed among 15.45%, followed by diabetic nephropathy in 7.27%, post infectious GN in 7.1%, renal vasculitis in 4.25% and hypertensive nephropathy in 1.52%. Among females, lupus was the most frequent pathology in that study and it corresponds to our findings [28]. However, another study done on the same settings in the period of 2015- 2018 found that MCD was the most common histopathological diagnosis (86/345) followed by lupus (20%), FSGS (14%), IgAN (12%), DN (10%) and MGN (9%) [27]. Therefore, these conflicting results in Sri Lanka should be further investigated to achieve unity.

Our findings were similar to the reports from other South Asian countries, America, Egypt, Spain and Iran, where the most common diagnosis was FSGS [13, 15–22, 35–38], Other studies from Europe, East Asia and Australia showed conflicting findings where they reported IgAN to be the most prevalent histopathological diagnosis [6–12, 14, 39–45]. Nevertheless, some studies in South Asia had different results as well, where they reported MCD [46, 47] and DPGN [29] as the most frequent diagnosis. Studies done in Thailand [48], Oman [49], Saudi Arabia [50], South Africa [51] and Serbia [30] showed lupus as the most common diagnosis (Table 6). Differences in the prevalence of histopathological diagnoses across nations could be due to the difference in socio demographic factors, environmental factors and difference in the healthcare systems across different countries.

**Table 6:** Epidemiology of renal biopsy from national registries and studies

**Region**	**Study**	**Country**	**Study period (years)**	**Number of cases (Female %)**	**Mean age** **(years)**	**Most prevalent glomerulonephritis in order**
South Asia	Present study	Sri Lanka	2018-2019	79 (55.2)	46.1 ±15.2	FSGS, Lupus, PSGN, DN, HTN, MCD, IgAN, IN, Vasculitis, Antibody mediated rejection, MGN
	Pilapitiya et al [27]	Sri Lanka	2012-2019	257 (33)	-	IgAN, FSGS, Primary chronic TIN, CINAC, Lupus, MCD, MPGN, DN, CresGN, DPGN, Acute TIN, MGN
	Basnayake et al [46]	Sri Lanka	2015-2018	345 (59.4)	-	MCD, Lupus, FSGS, IgAN, DN, MGN
	Basnayake et al [28]	Sri Lanka	2010-2019	2680 (50.3)	-	Lupus, FSGS, MCD, IgAN, DN, post infectious GN, MGN, Vasculitis, MPGN, HTN, Amyloidosis
	Islam et al [29]	Bangladesh	July -Dec 2015	235	31.93 ± 15.13	DPGN, FSGS, MPGN, Lupus, IgAN, MN, IgMN, TIN, DN, MCD, ATN
	Krishna et al [15]	India	2012-2015	270 (34.81)	31.48 ± 13.46	FSGS, DPGN, MN, MPGN, IgAN, Lupus, MCD, Mespgn, CSGN, CresGN, Vasculitis, DN, ATN, Amyloidosis, TMA, FNGN, IgMN, CIN
	Das et al [47]	India	1990–2008	1849 (41)	32.27 ± 18.37	MCD, Lupus, FSGS, MN, IgAN, Mespgn, DPGN, CresGN, IgAN, MPGN, CIN, ATN, Amyloidosis, DN, FNGN, Vasculitis, TMA
	Balkrishnan et al [16]	India	1990–2001	5016	-	FSGS, DPGN, MCD, MN, IgAN, Mespgn, Lupus, MPGN, DN, CIN, ATN, Amyloidosis
	Rathi et al [17]	India	2002-2007	364 (39.8)	31.5 ± 11	FSGS, MN, MPGN, MCD, Lupus, CSGN, Amyloidosis, DPGN, IgAN, DN
	Golay et al [18]	India	2010-2012	410 (42.19)	33.68 ± 13.88	FSGS, MCD, MN, MPGN, IgAN, Lupus, DPGN
	Mittal et al [35]	India	2006-2016	3275 (38.1)	33.2 ± 14.2	FSGS, MCD, MN, IgAN, Lupus, Amyloidosis
	Mubarak et al [19]	Pakistan	1995-2008	1793 (38.1)	32.9 ± 12.8	FSGS, MN, CSGN, ATN, MCD, CresGN, Lupus, Amyloidosis, CIN, IgMN, Mespgn, IgAN, FNGN, MPGN, DN, TMA
South East Asia	Parichatikanond et al [48]	Thailand	1982–2005	3555	-	Lupus, IgMN, IgAN, MGN
East Asia	Wang et al [32]	China	1996-2010	917 (45.72)	33.13 ± 14.13	Mespgn, IgAN, MCD, MN, Lupus, DN, MPGN, FSGS, DPGN, TMA, CresGN, CSGN, ATN, Vasculitis
	Sugiyama et al [6]	Japan	2007–2008	2126 (46.9)	44.9±21.5	IgAN, MGN, minor glomerular abnormalities, MesGN, DN
	Pan et al [7]	China	1997–2011	6337 (47.8)	33.6±18.0	IgAN, FSGS, MGN, MCD, MesGN
	Li LS, Liu ZH [8]	China	1979–2002	13519 (42.6)	32.7±12.2	IgAN, MesGN, Lupus, MGN, HSP, FSGS
	Bae et al [39]	Korea	1981–2010	2450 (43)	35.9 (15–91)	IgAN, MCD, MGN, MesGN, LN, FSGS
Middle East	Monfared et al [36]	Iran	2001-2006	336 (26.2)	40.12 ± 16.78	FSGS, MN, MCD, Lupus, CIN, MPGN, CresGN, CSGN, IgAN, Amyloidosis, ATN
	Al Riyami et al [49]	Oman	1992-2010	133 (63.9)	-	Lupus, FSGS, MN, CSGN, Mespgn, DN, DPGN, MPGN, Amyloidosis, ATN, CIN, CresGN, TMA, FNGN
	Ossareh et al [52]	Iran	1998-2007	1407 (45.8)	36.5 ± 15.5	MGN, IgAN, Lupus, FSGS, MCD, MPGN
	Naini et al [53]	Iran	1998-2001	462 (42.2)	33.6±15.7	MGN, IgAN, MPGN, Lupus, FSGS, MCD
	Mardanpour et al [54]	Iran	2007-2012	266 (51.5)	37.4±15.8	MCD, FSGS, MGN, Lupus, IgAN, MPGN
	Rahbar M. [55]	Iran	2003–2007	135 (65.7)	16.5	MCD, MGN, Lupus, FSGS, MPGN, IgAN
	Mohammadhoseiniakbari et al [37]	Iran	2006–2007	393 (45.3)	31.9±15.9	FSGS, MGN, Lupus, IgAN, MPGN, MCD
	Jafari et al [56]	Iran	2007–2009	130 (45)	32.98	MGN, Lupus, MPGN, IgAN, MCD
Eastern Mediterranean	Barsoum RS, Francis MR [38]	Egypt	1998–1999	1234 (45.9)	30.5±17.4	FSGS, MesGN, mesangiocapillary GN, MGN, MCD
	Karnib et al [57]	Lebanon	2003-2007	1048 (45.6)	36.7±20	MesGN, FSGS, benign nephroangiosclerosis, IgAN, tubulointerstitial nephropathy
	Huraib et al [50]	Saudi Arabia	-	1013	PGN: 30.8±15.1 SGN: 29.6±13.8	Lupus, FSGS, MPGN, MesGN (non-IgAN), MCD, MGN
Africa	Okpechi et al [51]	South Africa	2000–2009	1284 (54.8)	36.8±14.0	Lupus, infection-related GN (including HIV), vascular diseases, MCGN
	Okpechi et al [30] *(Systematic Review and Meta-Analysis)*	Africa	1980-2014	12093	-	MCD, FSGS, MCGN, CresGN, hepatitis B, Lupus, IgAN
America	Polito et al [20]	Brazil	1993–2007	9617 (51)	35.1±18.6	FSGS, MGN, IgAN, Lupus, MCD
	Arias et al [21]	Colombia	1998–2007	1040 (56.7)	28.2±17.6	FSGS, Lupus, IgAN, PIGN, MGN, MCD
	Nair R, Walker PD. [22]	USA	2001–2005	1228	-	FSGS, IgAN, MGN, MCD, MPGN
Europe	Horvatic et al [40]	Croatia	1997–2012	922 (42.2)	-	IgAN, FSGS, MGN, Hereditary nephritis, Pauci-immune glomerulonephritis
	Maixnerova et al [41]	Czech	1994–2011	10472 (42.2)	40.2	IgAN, LN, MGN, FSGS, MCD
	Heaf [58]	Denmark	1985–1997	2380 (41)	42.6±20.2	MesGN (including IgAN), MCD, FSGS, CresGN, MGN
	Simon et al [14]	France	1976–2002	1742	-	IgAN, MGN
	Molnár et al [10]	Hungary	2006-2020	1135	44.2 ± 21.9	IgAN, FSGS, MN, MCD, Lupus, MPGN
	Schena FP [11]	Italy	1987–1993	13835	-	IgAN, immune-mediated GN (including Lupus), MGN, FSGS
	Zaza et al [12]	Italy	1998–2010	4378 (37.9)	50.4±17.7	IgAN, MGN, FSGS
	Hanko et al [42]	Ireland	1976–2005	1844 (39)	49±17.8	IgAN, MGN, MPGN, MCD, FSGS
	Brazdziute et al [43]	Lithuania	1994–2012	3640 (41.6)	43.2±20.0	IgAN, FSGS, MPGN, CresGN, MCD
	Carvalho et al [44]	Portugal	1977–2003	2216 (50.1)	-	IgAN, Lupus, MesGN (non-IgAN), MCD, MGN
	McQuarrie et al [45]	Scotland	2002–2006	2480 (43.1)	55.6±1.3	IgAN, MGN, FSGS
	Naumovic et al [59]	Serbia	1987–2006	1626 (48.8)	39.1±13.8	Lupus, MesGN (non-IgAN), MGN, FSGS, IgAN, MPGN
	Rivera et al [13]	Spain	1994–2001	8722 (40)	-	FSGS, Lupus, MGN, IgAN, MesGN
	Covic et al [33]	Romania	1995-2004	308 (48.5)	-	MPGN, MesGN, FSGS, MGN, MCD, CresGN
	Volovăt et al [34]	Romania	2005-2010	239 (41.5)	41.9±2.8	MN, MPGN, FSGS, MesGN, MCD
	Hur et al [60]	Turkey	1996–2009	1702 (48)	40±15.3	Amyloidosis, Lupus, FSGS, MGN, IgAN
Oceania	Jegatheesan et al [9]	Australia	2002–2011	2048 (40)	48±17	IgAN, FSGS, DN, CresGN, Lupus, MGN
	Ling Goh et al [61]	Australia	2007-2020	364	52.7 ± 15.3	DN, FSGS, glomerulomegaly, IgAN, HTN, PIGN

*CINAC* Chronic interstitial nephritis in agricultural communities, *CresGN* Crescentic glomerulonephritis, *DN* Diabetic nephropathy, *DPGN* Diffuse proliferative glomerulonephritis, FNGN Pauci-immune focal necrotizing glomerulonephritis, *FSGS* Focal segmental glomerulosclerosis, *GN* Glomerulonephritis, *HIV* Human immunodeficiency virus, *HSP* Henoch–Schönlein purpura, *HTN* Hypertensive nephropathy, *IgAN* Immunoglobulin A nephropathy, *IgMN* Immunoglobulin M nephropathy, *IN* Interstitial nephritis, *MCD* Minimal change disease, *MCGN* Mesangiocapillary GN, *MesGN* Mesangioproliferative glomerulonephritis, *MGN* Membranous glomerulonephritis, *MPGN* Membranoproliferative glomerulonephritis, *MN* membranous nephropathy, *PGN* Primary glomerulonephritis, *PIGN* Post-infectious glomerulonephritis, *SGN* Secondary glomerulonephritis, *TIN* Tubulointerstitial nephritis, *TMA* Thrombotic Microangiopathy

In the present study the leading indication for biopsy was nephrotic range proteinuria and its most common pathologic diagnosis was FSGS (27.6%) followed by DN (17.1%), lupus nephritis (14.4%), MCD (11.8%), IgAN (9.2%) and HTN (7.8%). The most frequent diagnosis for nephrotic range proteinuria with hematuria was PSGN (30%) followed by FSGS (25%) and lupus nephritis (25%). Lupus nephritis was the most common diagnosis (40%) for sub nephrotic range proteinuria followed by HTN (20%), interstitial nephritis (15%), PSGN (10%) and DN (10%). The most common diagnosis in the sub nephrotic proteinuria with hematuria group was PSGN (69.2%) followed by lupus nephritis (23.1%) and FSGS (7.7%) (Table 1).

There were 36 (25.7%) diabetes patients in the sample and 19 patients had no morphologic signs of diabetic kidney disease in the renal biopsy. The most common pathologic diagnoses among patients with diabetes were DN (42.2%), FSGS (25%) and lupus (11.1%). Among the group without diabetes, the most common pathologies were lupus (23%) FSGS (21.1%), PSGN (21.1%), HTN (9.6%) and MCD (8.6%) (Table 5). However due to the relatively small sample size and absence of the comorbid duration, we did not divide the comorbid details into age categories and therefore we cannot comment on the above relationship between the age categories. Nevertheless, in our sample there were 77 patients below 50 years and 41 patients below the age of 40 years. This might be a partial explanation for these findings which needs future multicentric and a larger sample size to further investigate among the elderly and younger groups. Interestingly, a study done in Poland with 352 patients aged ≥65 years compared with a control group of 2214 individuals aged 18-64 years showed a prevalence of 18.2% diabetes patients among elderly individuals, and as much as 75% of them had no morphologic signs of diabetic kidney disease in the renal biopsy [31].

Though there were numerous papers published regarding frequency of indication and histopathological diagnosis of biopsy-proven kidney diseases, due to several factors our findings cannot be compared for conclusive interpretations. Firstly, time period of the studies conducted (the present study was from 2018-2019). Secondly, the age of the study participants (in our study the average age of the patient was 46 ±15.3 years and ranged from 18 - 76 years. Some studies evaluated cases of all ages, while some included only children, adults or the elderly. Moreover, the classification of age groups was not similar in different studies. Thirdly, the categorization of indications and type of biopsies varied in different studies. We studied all types of renal disorders as a whole, while some studies included only GN cases, whereas some included only native kidneys. In addition, some studies had categorization of primary GN, secondary GN, vascular disorders, hereditary and metabolic disorders. Fourthly, the lack of ability to generalize the data to the whole country since most studies were done in one or several centers in the country and only few countries had the capability of using a national registry [11, 21, 58]. Lastly, the different socioeconomic status of different countries.

It is evident from this study that similar indications are present with different renal abnormalities, thereby signifying renal biopsy as the most important diagnostic modality in renal disease, and majority of the cases are potentially treatable if diagnosed early. Therefore, it emphasizes the importance of maintaining a nationwide renal registry. This could help to identify the prevalence of various renal pathologies in different age groups, gender, geographies, socioeconomic classes that encounter in clinical practice in a developing nation like Sri Lanka. This may also help healthcare providers and nephrologists for early detection and improve treatment of renal pathologies. Nevertheless, despite the valuable insights provided by the study on the epidemiology of biopsy proven renal diseases in Sri Lanka, it is noteworthy that the sample size was small and the study period was relatively short (19 months). Hence, the results of this study cannot be considered definitive or conclusive. Therefore, we emphasize the importance of conducting multi-centric studies with longer study periods to gain a deeper understanding of the patterns of renal disease in Sri Lanka in the future.

Our study has several limitations. We used the most probable diagnoses and, in the sample, we did not have overlapping diagnoses. However, the lack of EM facility at the center was a limiting factor in determining co-existing diseases such as IGAN or FSGS. Relatively short duration of study resulting in a smaller sample size of only 140 patients is another key limitation which may not reflect the disease pattern. Due to the small sample size, we did not categorize the diagnoses such as primary and secondary GN. Though we have divided age groups within indication and diagnoses, we did not compare the association of indication to histopathological diagnoses within age groups as a result of the smaller sample size. In addition, our patient group is above 18 years and we cannot comment on the clinicopathological spectrum of renal diseases of children. Although this study was performed in a tertiary care hospital in an urban city of Sri Lanka, a significant proportion of patients come to the specialist clinics from outside the city. Therefore, we cannot conclude that the findings we obtained applies only to the local city population. Furthermore, since Sri Lanka is a multicultural, multilinguistic and multireligious country with varying education levels, our cohort may also not be fully representative of the spectrum of renal disease in Sri Lanka as a whole.

## Conclusions

In our study of 140 patients, nephrotic syndrome was the indication for renal biopsy in more than 50% of patients. The most common histological diagnosis was FSGS whereas the least frequent diagnosis reported was MGN. It is evident from this study that similar indications are present with different renal abnormalities, thereby signifying that there is no alternative to renal biopsy. Our data show the pattern of renal biopsy from a single center in Sri Lanka, which is an initial step in the understanding of the epidemiology of renal diseases in the nation. Our findings were different from previous reports in Sri Lanka and other countries, which can be possibly explained by the difference in geography, socioeconomic status, genetic and environmental factors. However due to the relative short time period it may not sufficiently reflect the disease pattern for definite conclusions. Therefore, further large-scale, multicentric studies should be carried out for a longer period to evaluate the survival rates of patients, and a national registry for renal biopsies should be established. Present data represent an important contribution to the epidemiology of renal diseases in Sri Lanka and providing a valuable comparison with other renal biopsy registries worldwide, as a basis for nephrologists and health care providers to stimulate new analyses and improve treatment of renal diseases.

## Data Availability

The dataset is available with the Primary Investigator, Dr. Arjuna Marasinghe and can be provided upon request.

## Abbreviations

CINAC: Chronic interstitial nephritis in agricultural communities
CresGN: Crescentic glomerulonephritis
DN: Diabetic nephropathy
DPGN: Diffuse proliferative glomerulonephritis
FNGN: Pauci-immune focal necrotizing glomerulonephritis,
FSGS: Focal segmental glomerulosclerosis
GN: Glomerulonephritis
HIV: Human immunodeficiency virus
HSP: Henoch–Schönlein purpura
HTN: Hypertensive nephropathy
IgAN: Immunoglobulin A nephropathy
IgMN: Immunoglobulin M nephropathy
IN: Interstitial nephritis
MCD: Minimal change disease
MCGN: Mesangiocapillary GN
MesGN: Mesangioproliferative glomerulonephritis
MGN: Membranous glomerulonephritis
MPGN: Membranoproliferative glomerulonephritis
MN: Membranous nephropathy
PGN: Primary glomerulonephritis
PIGN: Post-infectious glomerulonephritis
SGN: Secondary glomerulonephritis
TIN: Tubulointerstitial nephritis
TMA: Thrombotic Microangiopathy
